# Aberrant T Helper 17 Cells and Related Cytokines in Bone Marrow Microenvironment of Patients with Acute Myeloid Leukemia

**DOI:** 10.1155/2013/915873

**Published:** 2013-08-18

**Authors:** Tian Tian, Shuang Yu, Min Wang, Cunzhong Yuan, Hua Zhang, Chunyan Ji, Daoxin Ma

**Affiliations:** ^1^Department of Hematology, Qilu Hospital, Shandong University, 107 West Wenhua Road, Jinan 250012, China; ^2^Department of Obstetrics and Gynecology, Qilu Hospital, Shandong University, Jinan 230001, China; ^3^Institute of Basic Medicine, Shandong Academy of Medical Sciences, Jinan 230001, China

## Abstract

In this study, we mainly investigate the role of Th17 cells, Th1 cells, and their related cytokines in the pathophysiology of AML. BM and PB were extracted from ND, CR, and relapsed-refractory AML patients and controls. Th subsets frequencies were examined by flow cytometry. BM plasma Th-associated cytokines levels were determined by ELISA. The frequencies of Th17 and Th1, and IFN-**γ** or TGF-**β** concentrations were significantly decreased in ND compared with CR patients or controls. Th17 percentage was significantly lower in BM than in PB for ND patients but was higher in BM for CR patients. However, in CR or relapsed-refractory patients, Th1 percentage in BM was higher than that in PB. Moreover, BM IL-17A level showed a decreased trend in ND patients. A significant elevation of plasma IL-6 level was found in ND compared with CR patients or controls. IL-17A showed the positive correlation with IL-6 concentration. And Th17 cells frequencies and TGF-**β**1 concentration were increased in BM from AML patients achieving CR after chemotherapy. Moreover, a significant decrease of BM plasma TGF-**β**1 level was found in M3 patients compared with the other subtypes. Our findings suggest that Th17 and related cytokines may be implicated in AML pathogenesis.

## 1. Introduction

Acute myeloid leukemia (AML) is a life-threatening hematopoietic stem cell neoplasm characterized by increased number of myeloid cells in the bone marrow and an arrest in their maturation, frequently resulting in fatal infection, bleeding, or organ infiltration, with or without leukocytosis [1–3]. The etiology of AML is heterogeneous and complex, but it is widely accepted that both environmental and genetic factors play significant roles in the development of AML. Immune system disorders have increased our understanding of leukemogenesis [4]. However, little is known about the immunopathological events, especially the abnormal T helper (Th) subsets, leading to the initiation and progression of this disease.

A novel IL-17-producing Th subset, termed Th17 cells, has been described in recent years [5–7]. In mice, the differentiation of Th17 cells is driven primarily by the cytokines transforming growth factor- (TGF-) *β* and IL-6, and it is known that IL-23 is necessary for the pathogenicity of Th17 cells [5, 8–10]. Retinoic acid-related orphan nuclear receptor gamma t (ROR-*γ*t) is a transcription factor that is considered to be important for the initiation and maintenance of Th17 cell lineage [11, 12] and regulating the differentiation of Th17 subset. However, less is known in human than in mice. Volpe et al. [13] have reported that TGF-*β*, IL-23, and proinflammatory cytokines (IL-1*β* and IL-6) were all essential for human Th17 differentiation. Nevertheless, Acosta-Rodriguez et al. [14] found that for human naive CD4^+^ T cells, ROR-*γ*t expression and Th17 polarization were induced by IL-1*β* and enhanced by IL-6 but were suppressed by TGF-*β* and IL-12.

Accumulating evidence demonstrated that Th17 cells play critical roles in several animal models of autoimmunity, such as experimental allergic encephalomyelitis (EAE) [15] and murine arthritis models [16, 17]. Besides, Th17 cells are considered to be involved in many human inflammatory diseases, including multiple sclerosis, psoriasis, and inflammatory arthritis [18–21]. What is more, Th17 cells and IL-17 have a regulatory role in normal hematopoiesis [22]. It has been established that Th17 cells participate in solid tumors [23]; however, the specific role of Th17 in cancer is debatable. Results from two studies in prostate and ovarian cancer patients suggested both beneficial and harmful implications of Th17 cells in tumor development [24, 25], while another ovarian cancer research [26] showed that Th17 may provide protection to human tumor immunity through inducing Th1-type chemokines and recruiting effector cells to the tumor microenvironment. Recently, Zhang et al. [27] showed a prominently increased frequencies of Th17 and IL-17 level in patients with uterine cervical cancer and cervical intraepithelial neoplasia. However, studies about circulating Th17 cells in AML are divergent. Wu et al. showed that Th17 frequency was significantly increased in the peripheral blood of patients with AML compared with controls [28], while Fan et al. demonstrated that Th17 frequencies and IL-17A levels in ND and CR AML patients were lower than healthy controls [29]. Up to now, there was no study about Th17 cells in bone marrow (BM) microenvironment of AML. Considering the important role of BM microenvironment in the hematopoiesis and the formation of the primitive cells, it is necessary to do further researches to interpret the specific role of Th17 cells in BM microenvironment of AML.

In this study, we examined the frequencies of Th17 and Th1 cells and the concentrations of related cytokines (IL-17A, IL-6, TGF-*β*1, and IFN-*γ*) in BM or PB of patients with different AML stages and controls and evaluated their involvement in the pathogenesis and progression of AML.

## 2. Materials and Methods

### 2.1. Patients and Controls

Forty-nine newly diagnosed (ND) (23 females and 26 males; age range, 21–83 years; median age, 43 years), 18 relapsed-refractory (8 females and 10 males; age range, 18–63 years; median age, 41 years), and 38 complete remission (CR) AML patients (19 females and 19 males; age range, 19–71 years; median age, 45 years) were enrolled in this study. AML patients were diagnosed according to the French-American-British (FAB) classification system [30]. CR was defined based on International Working Group Criteria [31]. Relapsed-refractory patients failed to achieve CR after two courses of standard induction chemotherapy or relapsed in 6 months after the first CR. Because bone marrow aspiration is a quite invasive procedure, individuals with slight iron deficiency anemia, having no immunological changes, were used as controls. The control group consisted of 19 individuals (14 females and 5 males; age range, 18–67 years; median age, 39 years). Participants' characteristics were provided in Table 1. This study was approved by the Medical Ethical Committee of Qilu Hospital, Shandong University, China. Informed consent was obtained from all patients before enrollment in the study in accordance with the Declaration of Helsinki.

### 2.2. Treatment Regimen

Newly diagnosed patients with acute promyelocytic leukemia (APL, subtype M3) received all-trans retinoic acid with or without concurrent induction chemotherapy. Newly diagnosed patients with non-M3 AML subtypes underwent standard induction chemotherapy with one of the anthracyclines (doxorubicin or idarubicin) for 3 days and cytarabine for 7 days and received consolidation therapy with high-dose cytarabine with or without the anthracycline after achieving CR. 

### 2.3. Bone Marrow and Peripheral Samples

BM and PB samples from different stages of AML patients and controls were collected in heparin-anticoagulant vacutainer tubes. Plasma of BM was obtained after centrifugation and stored at −80°C for measurement of cytokine levels.

### 2.4. Flow Cytometric Analysis of Th17 and Th1 Cells

Intracellular cytokines were studied by flow cytometry to reflex the cytokine-producing cells. Briefly, heparinized whole BM (400 uL) with an equal volume of Roswell Park Memorial Institute (RPMI)-1640 medium was incubated for 4 h at 37°C in 5% CO_2_ in the presence of 2.5 ng/mL of phorbol myristate acetate (PMA), 1 mg/mL of ionomycin, and 1.7 mg/mL of monensin (all from Alexis Biochemicals, San Diego, CA, USA). PMA and ionomycin are pharmacologic T-cell-activating agents that mimic signals generated by the T-cell receptor (TCR) complex and have the advantage of stimulating T cells of any antigen specificity. Monensin is used to block the intracellular transport mechanisms, thereby leading to an accumulation of cytokines in the cells. After incubation, the cells were stained with Alexa Fluor 647 anti-human CD4 monoclonal antibody at room temperature in the dark for 20 min. The cells were next stained with PerCP/Cy5.5 anti-human IL-17A or anti-human IFN-*γ* monoclonal antibody after fixation and permeabilization. All antibodies were obtained from BioLegend (San Diego, CA, USA). Isotype controls were utilized to enable correct compensation and to confirm antibody specificity. Stained cells were analyzed by flow cytometric analysis using a FACS Calibur cytometer equipped with CellQuest software (BD Bioscience Pharmingen, San Jose, CA, USA). Considering the relatively less number of lymphocytes in BM microenvironment of AML patients, we circled and collected 5000 CD4^+^ cells during the step of FACS cell collection. For analysis, we first gated CD4^+^ lymphocytes, then analyzed the proportion of Th17 (CD4^+^IL-17^+^) and Th1 (CD4^+^IFN-*γ* ^+^) cells in CD4^+^ lymphocytes.

### 2.5. IL-17, TGF-*β*1, IL-6, and IFN-*γ* Enzyme-Linked Immunosorbent Assay (ELISA)

BM plasma Th17-related cytokines (IL-17A, total TGF-*β*1, and IL-6) and IFN-*γ* levels were determined using ELISA method according to the manufacturer's recommendations (lower detection limit 0.5 pg/mL, 9 pg/mL, 0.92 pg/mL, 0.99 pg/mL, resp.; all ELISA kits are from eBioscience).

### 2.6. Statistical Analysis

Results were expressed as mean ± SD or median (range). Statistical significance among patients in the four groups was determined by ANOVA, and difference between two groups was determined by Newman-Keuls multiple comparison test (*q* test) unless the data were not normally distributed, in which case Kruskal-Wallis test (*H* test) and Nemenyi test were used. The Pearson or Spearman correlation test was used for correlation analysis depending on data distribution. *P* value < 0.05 was considered statistically significant.

## 3. Results

### 3.1. Abnormal Th17 Cells in AML Patients

**Figure 1:**
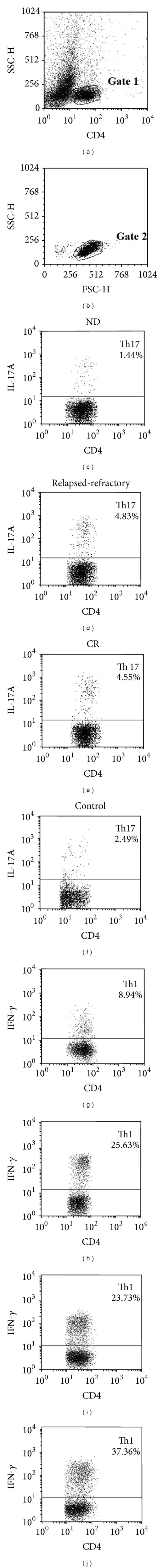
BM Th17 and Th1 cells in representative patients with ND, CR, and relapsed-refractory AML and controls. (a) 5000 CD4^+^ cells were collected by flow cytometry. (b) CD4^+^ lymphocytes were gated. (c), (d), (e), and (f) The percentages of BM Th17 (CD4^+^ IL-17^+^) cells in ND, CR, and relapsed-refractory AML patients and controls. (g), (h), (i), and (j) The percentages of BM Th1 (CD4^+^ IFN-*γ* ^+^) cells in ND, CR, and relapsed-refractory AML patients and controls.

**Figure 2:**
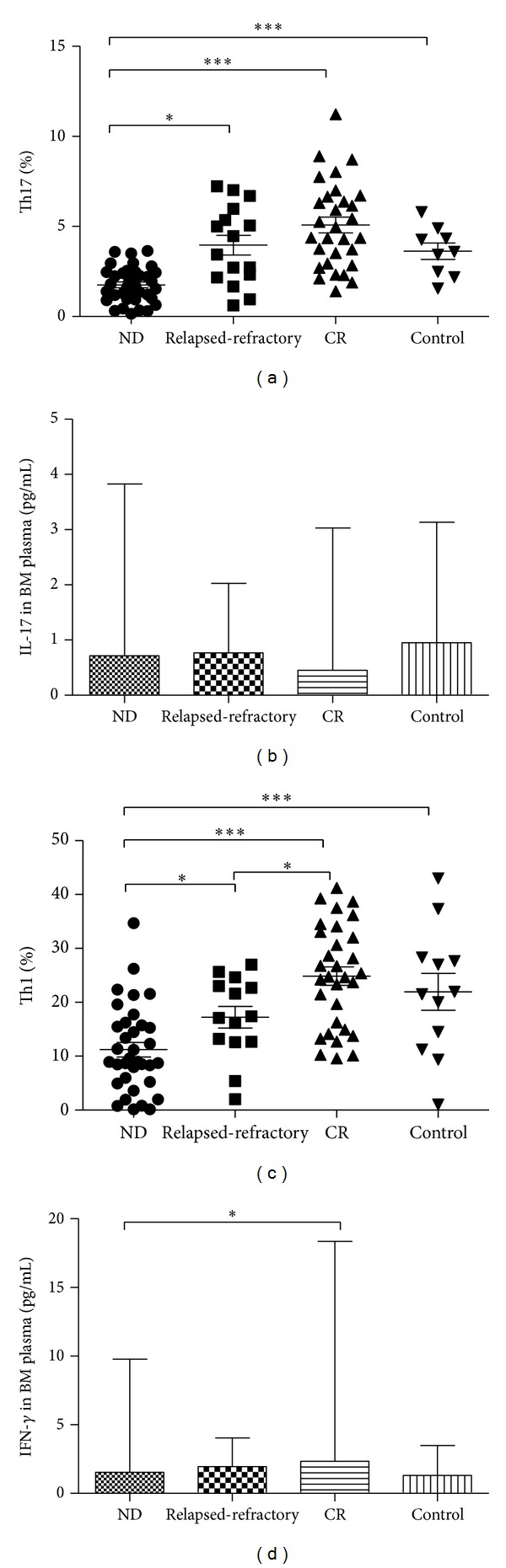
Th subsets and their related cytokines in ND, relapsed-refractory, and CR AML patients and controls. (a) The percentage of BM Th17 cells was significantly decreased in ND AML patients compared with CR patients or controls after stimulation with phorbol myristate acetate, ionomycin, and monensin for 4 h. (b) The level of BM plasma IL-17A showed the decreased trend in the ND, relapsed-refractory, or CR AML patients compared with controls, though no statistical significance exists. (c) The percentage of BM Th1 cells was significantly decreased in ND AML patients compared with relapsed-refractory or CR patients or controls. (d) The level of BM plasma IFN-*γ* was decreased in the ND AML patients compared with CR patients.

We analyzed the frequency of Th17 cells based on cytokine patterns after in vitro activation by PMA plus ionomycin in short-term culture. The expression of a typical dot-plot of Th17 cells in representative ND, relapsed-refractory, CR AML patients, and controls was shown in Figure 1. In different stages of AML, Th17 cells frequencies were statistically decreased in ND patients (1.76 ± 0.96%) compared to CR (5.082 ± 2.4%; ***P < 0.0001) or relapsed-refractory AML patients (3.97 ± 2.17%; *P = 0.0011) or controls (3.63 ± 1.37%; ***P < 0.0001) (Figure 2(a)). Compared with BM plasma IL-17A level in controls (median, 0.95 pg/mL; range, 0.19–3.14 pg/mL), though there was a decreased trend in ND (median, 0.72 pg/mL; range, 0.19–3.83 pg/mL), relapsed-refractory (median, 0.77 pg/mL; range, 0.14–2.03 pg/mL), or CR (median, 0.45 pg/mL; range, 0.14–3.03 pg/mL) AML patients, no statistical significance was observed (Figure 2(b)). No significant correlation was found between Th17 and plasma IL-17A levels in all the groups.

**Figure 3:**
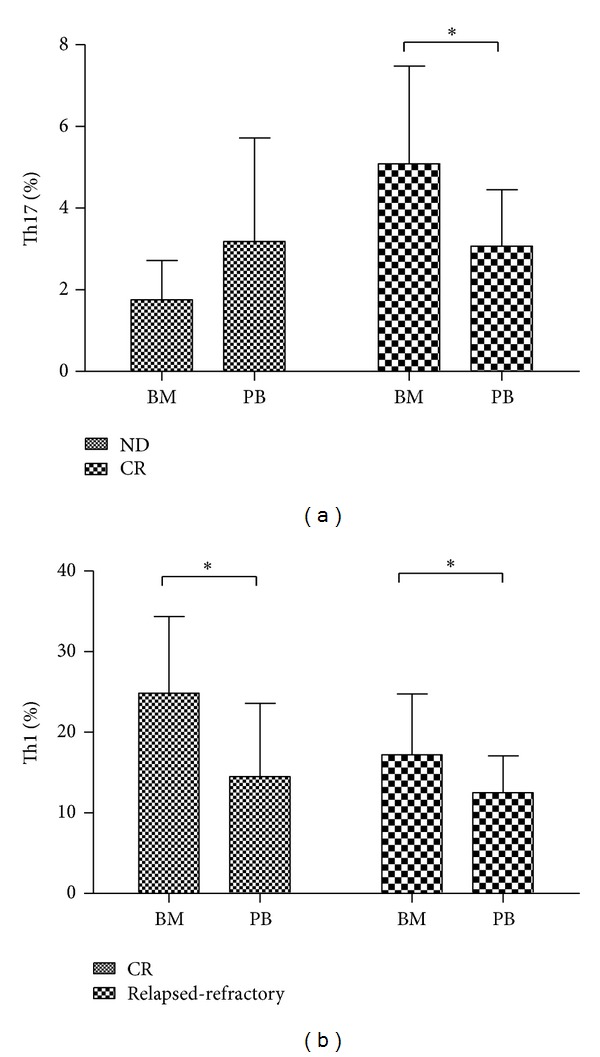
The comparison of Th17 or Th1 cells in BM and PB in different stages of AML patients. (a) Th17 cells percentage was markedly higher in BM than in PB in CR AML patients. (B) Th1 cells percentage was significantly increased in BM compared with in PB of CR or relapsed-refractory patients.

Meanwhile, we compared the Th17 cells between BM and PB in AML patients. The results showed that in ND AML patients, Th17 percentage was lower in BM (1.76 ± 0.96%) than in PB (3.18 ± 2.53%), but did not reach the statistical difference (*P* = 0.08). In CR patients, Th17 percentage was markedly higher in BM (5.08 ± 2.4%) than in PB (3.07 ± 1.38%; *P = 0.0005) (Figure 3(a)).

### 3.2. Abnormal Th1 Cells in AML Patients

We also analyzed Th1 frequencies in different stages of AML patients. The expression of a typical dot-plot of Th1 cells was shown in Figure 1. In different stages of AML, Th1 frequencies were statistically decreased in ND patients (11.22 ± 7.99%) compared to CR (24.84 ± 12.72%; ***P < 0.0001) or relapsed-refractory AML patients (17.23 ± 7.52%; *P = 0.02) or controls (21.95 ± 11.86%; *P = 0.001) (Figure 2(c)). Moreover, Th1 percentage was markedly decreased in relapsed-refractory stage than in CR stage (*P = 0.01). Compared with CR (median, 2.34 pg/mL; range, 1.48–18.37 pg/mL) patients, a significant decreased BM plasma IFN-*γ* level was found in ND AML patients (median, 1.55 pg/mL; range, 0.1–9.77 pg/mL; *P = 0.03) (Figure 2(d)).

Meanwhile, we compared the Th1 cells between BM and PB. The results showed that Th1 percentage was significantly higher in BM (24.84 ± 9.51%; 17.23 ± 7.52%, resp.) than in PB (14.51 ± 9.08%; 12.52 ± 4.56%, resp.) in CR (*P = 0.0011) or relapsed-refractory patients (*P = 0.049) (Figure 3(b)).

### 3.3. Increased IL-6 and Decreased TGF-*β*1 Concentration in Plasma from ND AML Patients

**Figure 4:**
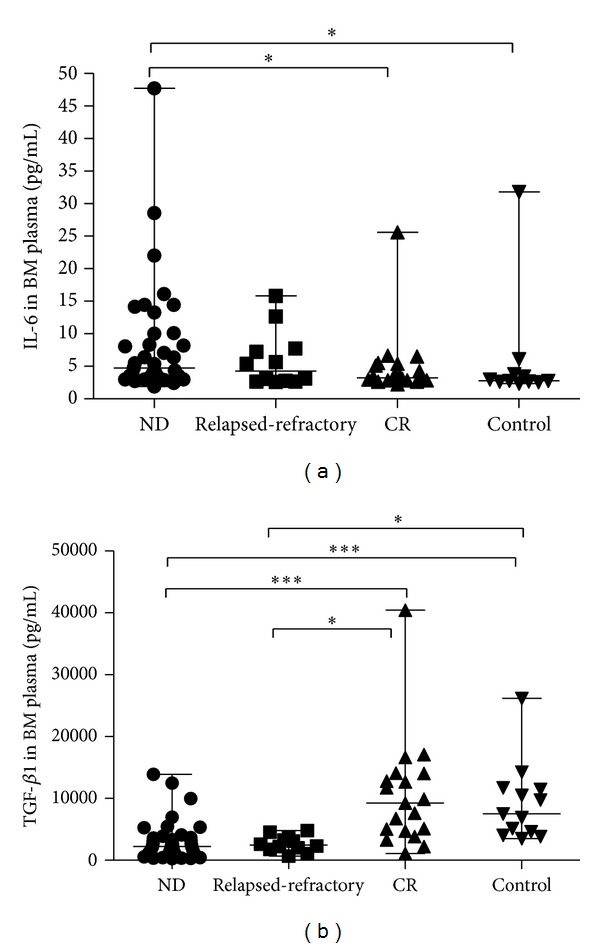
Th17-related cytokines in AML patients and controls. (a) The level of IL-6 was significantly higher in ND AML patients than in CR patients or controls (*P < 0.05). (b) A statistical decrease of plasma TGF-*β*1 level in ND or relapsed-refractory AML patients was found compared with CR patients or controls (*P < 0.05, ***P < 0.0001).

Concentrations of bone marrow plasma IL-6 and TGF-*β*1 were measured by ELISA. For IL-6 level, there was a significant increase in ND patients (median, 5.34 pg/mL; range, 1.92–175.66 pg/mL) compared to controls (median, 2.78 pg/mL; range, 2.36–31.81 pg/mL; *P = 0.0071) or CR patients (median, 3.25 pg/mL; range, 2.63–25.62 pg/mL; *P = 0.01). Moreover, we observed an increased trend in relapsed-refractory patients (median, 4.25 pg/mL; range, 2.60–15.83 pg/mL) compared with controls or CR patients (Figure 4(a)).

For TGF-*β*1, the level was markedly decreased in ND (median, 2222.95 pg/mL; range 289.8–13883.7 pg/mL) and relapsed-refractory AML patients (median, 2462.35 pg/mL; range, 677.2–4799.8 pg/mL) compared with CR patients (median, 9273.68 pg/mL; range, 1092.8–40438.5 pg/mL; ***P < 0.0001; *P = 0.0003; resp.) or controls (median, 7510.33 pg/mL; range, 3510–26205.9 pg/mL; ***P < 0.0001; *P = 0.0002, resp.) (Figure 4(b)).

### 3.4. Correlation between Th17 Cells and Related Cytokines in BM Environment of ND AML Patients

**Figure 5:**
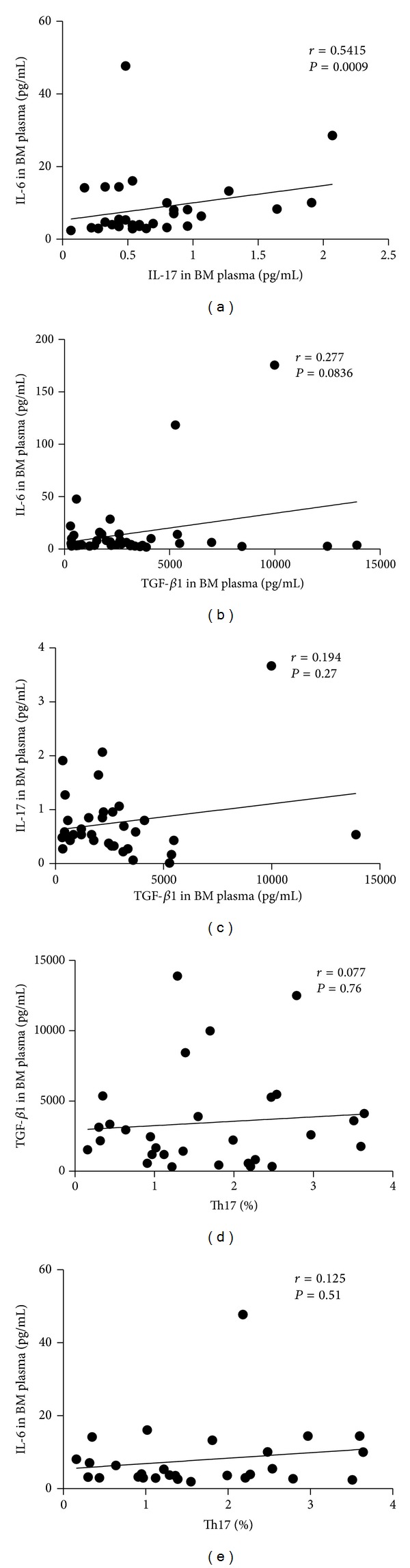
Correlations between the related cytokines in ND AML patients. (a) A positive correlation was found between IL-6 and IL-17A concentration (*r* = 0.5415, *P* = 0.0009) in ND AML patients. (b) Marginal correlation existed between TGF-*β*1 and IL-6 concentration (*r* = 0.277, *P* = 0.0836). (c, d, e) No significant correlation was found between TGF-*β*1 and IL-17A, Th17 and TGF-*β*1, or Th17 and IL-6.

Our research failed to show any statistical correlation between Th17 and IL-17A (*r* = 0.04, *P* = 0.86) or IL-6 (*r* = 0.125, *P* = 0.51) or TGF-*β*1 (*r* = 0.077, *P* = 0.76) concentration (Figures 5(d) and 5(e)). However, it demonstrated that IL-17 concentration showed a positive correlation with the level of IL-6 (*r* = 0.5415, *P* = 0.0009) (Figure 5(a)) in ND AML patients, but did not reach statistical correlation in CR, relapsed-refractory, or control groups. There was marginally statistical correlation between TGF-*β*1 and IL-6 concentrations (*r* = 0.277, *P* = 0.0836) (Figure 5(b)). No significant correlation was found between TGF-*β* and IL-17A (*r* = 0.194, *P* = 0.27) (Figure 5(c)).

### 3.5. Elevated Frequencies of Th17 Cells in BM from AML Patients Achieving CR after Chemotherapy

**Figure 6:**
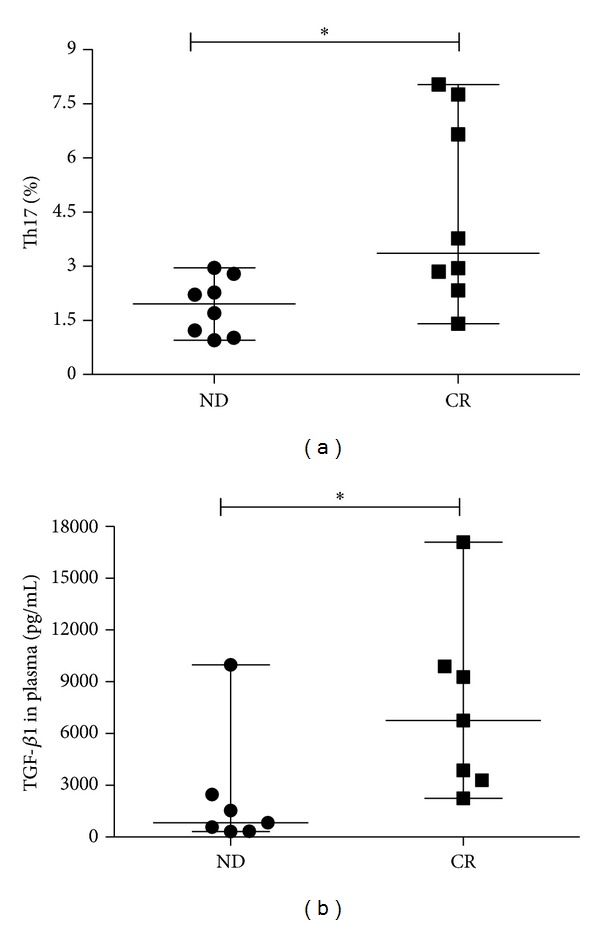
Th17 and associated cytokines in the same AML cohort. (a) Th17 percentage was observed significantly lower in ND stage than in CR stage of the same AML patients (*P < 0.05). (b) A markedly lower TGF-*β*1 concentration was observed in ND stage than in CR stage of the same AML patients (*P < 0.05).

To further understand the influence of chemotherapy on AML BM microenvironment, we observed the whole treatment process in 9 AML patients. CR was obtained after the standard induction chemotherapy. After chemotherapy, Th17 frequencies were markedly increased (median, 3.36%; range, 1.41–8.03% versus median, 1.96%; range, 0.95–2.79%; *P = 0.0148) (Figure 6(a)). Moreover, plasma TGF-*β*1 level was also significantly elevated in CR stage (median, 6753.6 pg/mL; range, 2249.5–17092.3 pg/mL versus median, 832.7 pg/mL; range, 315.5–9979.6 pg/mL; *P = 0.0298) (Figure 6(b)).

### 3.6. BM Plasma TGF-*β*1 Level among Different FAB Subtypes of AML

**Figure 7:**
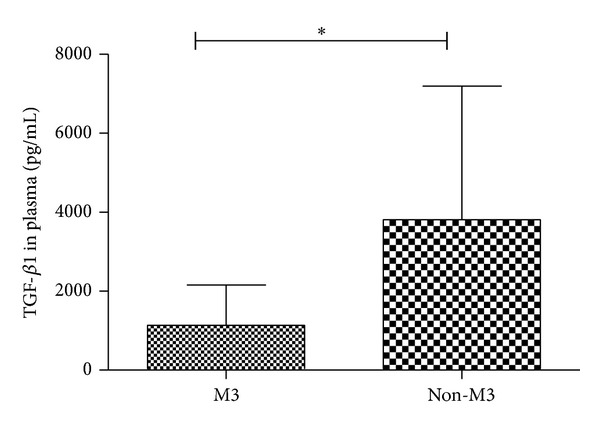
BM plasma TGF-*β*1 level among different FAB subtypes of AML. A significant decrease of BM plasma TGF-*β*1 level in M3 patients compared with the other subtypes (*P = 0.0005).

Because of different characteristics for various AML subtypes, we compared the Th17 subset and related cytokines in M3 with the other subtypes. We found a significant decrease of BM plasma TGF-*β*1 level in M3 patients compared with the other subtypes (1137.4 ± 1016.3 pg/mL versus 3809 ± 3388.6 pg/mL, *P = 0.0005) (Figure 7). We did not obtain the statistical difference of the other Th cells and related cytokines between these two groups.

## 4. Discussion

In this study, we firstly observed that aberrant Th17 or Th1 subset and associated cytokines in BM microenvironment are involved in AML pathogenesis, and chemotherapy partly ameliorates this turmoil. 

Th17 and their effector cytokines are being recognized as important mediators in autoimmune and inflammatory diseases, and our studies had demonstrated that Th17 cells were elevated in idiopathic thrombocytopenia (ITP) patients [32]. Previous studies had investigated Th17 cells in both murine and human solid tumors. However, the nature and the role of Th17 cells in cancer immunity remained elusive. In peripheral blood, Kryczek et al. [25] reported that the levels of Th17 were significantly increased both in prostate-tumor-bearing mice and epithelial ovarian carcinomas patients. Another researches in gastric cancer, uterine cervical cancer patients [27, 33] also found significantly elevated frequencies of Th17 cells in peripheral blood as well as in tumor draining lymph nodes. In blood malignant disease, our research showed that Th17 frequencies were increased in early-stage myelodysplastic syndrome (MDS) compared with the late-stage MDS or controls [34]. About the specific role of Th17 cells in peripheral blood of AML patients, the results were controversial, and so far no research was investigated in BM microenvironment of AML patients. Consistent with Fan et al.'s research that Th17 frequencies and IL-17A levels were significantly decreased in peripheral blood of ND and CR AML patients [29], our results demonstrated that Th17 cells were markedly decreased in BM microenvironment of ND patients compared with CR, relapsed-refractory patients or controls. What is more, the IL-17A in BM plasma showed a decreased trend in ND and relapsed-refractory AML patients. The possible explanation is that in addition to Th17 cells, other subset of T cells, including CD8^+^ T, NK T, and TCR*γδ* cells, have been demonstrated to produce IL-17A. Even non-T cells, such as neutrophils and lymphoid tissue inducer-like cells, can also be an innate source of IL-17A [35]. Our result in the same cohort of AML patients showed that decreased Th17 frequencies were partly corrected after standard chemotherapy, indicating the importance of Th17 in AML pathogenesis and measurement of Th17 frequencies may be valuable for evaluating therapeutic effect. In light of these results, the decrease of Th17 cells in ND AML patients may be explained as a depressed immune response, and the elevation of Th17 in the CR patients indicated a protective reaction of the immune system accompanied by the chemotherapy. In any case, Th17 cells may participate in the progression of AML. These results also suggest that the number of Th17 cells may relate to tumor burden, which may be as a prognostic target. Obviously, further researches are needed to explain the specific role of Th17 in AML patients.

Moreover, considering the decreased Th17 in BM compared with in PB of the ND AML patients; the possible interpretation is that leukemic cells inhibit the production and differentiation of normal immune cells, such as Th17 cells, which causes the lower percentage of Th17 cells in BM of ND AML patients. In CR AML patients, the leukemia cells were decreased greatly which results in the decreased inhibition, and Th17 cells percentage was elevated in BM than in PB.

Our result showed that Th1 cells were significantly decreased in ND or relapsed-refractory patients compared with CR or controls, which was consistent with the reduced immune function in AML patients. We also found that Th1 percentage was higher in BM than in PB from CR or relapsed-refractory patients. And Th1 cells showed the high trend in BM than in PB in ND patients, even not reaching the statistical significance. All these results indicated that Th1 may be a therapeutic target for AML patients. Further researches are needed to specify the role of Th1 subset in AML.

 To date, the knowledge of Th17 differentiation originates from experimental animals, whereas very little information exists about human Th17 cells. In mice, TGF-*β* is the cytokine critical for Th17 initiation, and IL-6 acts as a critical cofactor for Th17 cell differentiation [5, 8, 9]. In human being, the opinions about the role of TGF-*β* or IL-6 in Th17 cells differentiation were divergent. Our results showed that concentration of IL-6 was markedly elevated whereas TGF-*β*1 was significantly decreased in ND AML patients. Considering the decreased frequencies of Th17 in ND patients, we speculated that TGF-*β*1 may play a central role in the Th17 differentiation, which was consistent with three independent reports that TGF-*β* was critical for human Th17 cell differentiation [13, 36, 37]. Simultaneously, in ND AML patients, IL-6 concentration showed a positive correlation to the IL-17A levels, and a marginal correlation with TGF-*β*1, which may show that IL-6 and TGF-*β*1 cytokines coordinately promote the differentiation of Th17 cells and generation of IL-17A. Moreover, the variation of the Th17-related cytokines implies that they may play a pathological or protective role in AML process, or work as an effector cytokine. However, we must mention a caveat that TGF-*β*1 is produced in a latent form, and needs to be activated in order to exert its action. The active form of TGF-*β*1 is unstable. Therefore, it is always difficult to correlate TGF-*β*1 serum levels with biological outcomes in vivo.

Recently, more and more evidence supports deregulated TGF-*β*1 signaling in leukemogenesis, especially in M3. Our study showed that TGF-*β*1 was lower in M3 patients than in other subtypes. The results were consistent with a study that combined treatment with TGF-*β*1, and 1,25-dihydroxyvitamin D3 can cause terminal monocytic maturation in monocytic leukemic cell lines [38]. These all indicate the therapeutic potential of TGF-*β*1 in M3, even in all AML patients.

In summary, the downregulation of Th17 cells, Th1 cells, TGF-*β*1, and the secreted cytokine IL-17A and the upregulation of IL-6 concentration showed a strong relationship with AML activity. And standard induction chemotherapy may partly ameliorate the abnormal changes. All these results open a new avenue in the study of tumor immunotherapy. Further studies are awaited to clarify their specific roles in the pathophysiology of AML occurrence and development, finally providing the perspective for clinical treatment.

## Figures and Tables

**Table 1:** The characteristics of subjects.

	ND AML patients (*n* = 49)	CR AML patients (*n* = 38)	Relapsed-refractory AML patients (*n* = 18)	Controls (*n* = 19)
Age (years)	21–83	19–71	18–63	18–67
Gender (male/female)	26/23	19/19	10/8	5/14
WBC (∗10^9^/L)	31.06 ± 53.64	5.30 ± 2.13	40.12 ± 61.02	6.5274 ± 2.42
BM leukemic blast (%)	72.61 ± 40.91	1.17 ± 1.15	58.27 ± 29.13	
FAB subtype				
M1	2	0	0	
M2	7	2	1	
M3	9	14	1	
M4	9	9	5	
M5	22	13	11	

ND: newly diagnosed; CR: complete remission; WBC: white blood cell; FAB: French-American-British; BM: bone marrow.
